# Biodiversity and Structure of Microbial Community in Glacial Melts and Soil in the High Arctic Ny-Ålesund, Svalbard

**DOI:** 10.3390/microorganisms10101941

**Published:** 2022-09-29

**Authors:** Fang Zhang, Fenglin Lv, Mianrun Chen

**Affiliations:** 1 MNR Key Laboratory for Polar Science, Polar Research Institute of China, Shanghai 200136, China; 2 China Laboratory of Fisheries Oceanography, College of Fisheries, Ocean University of China, Qingdao 266071, China; 3 Southern Marine Science and Engineering Guangdong Laboratory (Zhuhai), Zhuhai 519000, China; 4 South China Sea Institute of Planning and Environmental Research, SOA, Guangzhou 510300, China

**Keywords:** prokaryotes, eukaryotes, glacial-related water, coastal water, unique microbial species, environmental correlations

## Abstract

Ny-Ålesund in Svalbard is a complex area with both continental and tidal glaciers. There are a lot of studies on prokaryotic and eukaryotic communities in coastal water and soil, but without studies in glacial-related waters. We make a distinctive and consolidated study on the structure of the prokaryotic and eukaryotic communities of pure glacial meltwater, glacial melting lake, glacial meltwater flowing via different types of soil at various elevations, estuarine glacial water and marine water. Moreover, we analyze the environmental–microbial relationships of the prokaryotic and eukaryotic communities via a canonical correspondence analysis and redundant analysis compared by a Pearson analysis. We found that there were distinct microbes in different environments. Altitude had significant correlations with prokaryotic and eukaryotic species in the 12 water samples (*p_pro_* = 0.001, *n_pro_* = 1010, and *p_euk_* = 0.012, *n_pro_* = 1651) (Pearson analysis). Altitude, temperature and salinity, respectively, accounted for 28.27%, 10.86% and 8.24% in the prokaryotic community structure and 25.77%, 17.72% and 3.46% in the eukaryotic, respectively, in water. Nitrogen, silicate and pH accounted for 38.15%, 6.15% and 2.48% in the prokaryotic community structure in soil and 26.65%, 12.78% and 8.66% in the eukaryotic. Eukaryotes were more stable than prokaryotes in changing environments. Cyanobacteria and dinoflagellates better adapt to a warming environment. Gammaproteobacteria and Chrysophysceae were most abundant in soil. Alphaproteobacteria, Bacteroidia, Mamiellophyceae and Prasinophytae were most abundant in water. Within these microbes, Bacilli and Chlorophyceae were only found in glaciers; Actinobacteria, KD94-96, Thermleophilia, Embryophyta, Trebouxiophyceae and Sordariomycetes were unique to soil.

## 1. Introduction

**Figure 1:**
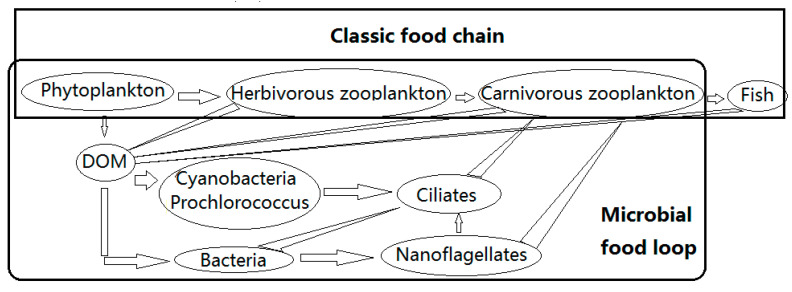
Microbial food loop and classic food chain [3].

Microorganisms are the basis of ecosystems and are the main components of the marine micro-food loop (Figure 1). They are small (<200 μm) but perform great functions within the ecosystem. Viruses, prokaryotes, archaea, microalgae, fungi, protozoa, etc. are the main components [1]. They can be producers, consumers and decomposers. Microorganisms are ubiquitous in different environments, as they are “Everything in Everywhere, but, the environment selects” [1,2]. Microorganisms are responsible for many important processes, including nutrient mineralization (bacteria) and dimethyl sulfoniopropionate (DMSP) production (phytoplankton named *Phaeocystis* and other ice algae and phytoplankton). All microorganisms produce greenhouse gas carbon dioxide (CO_2_), and some produce methane (CH_4_) [3,4,5,6,7,8].

**Figure 2:**
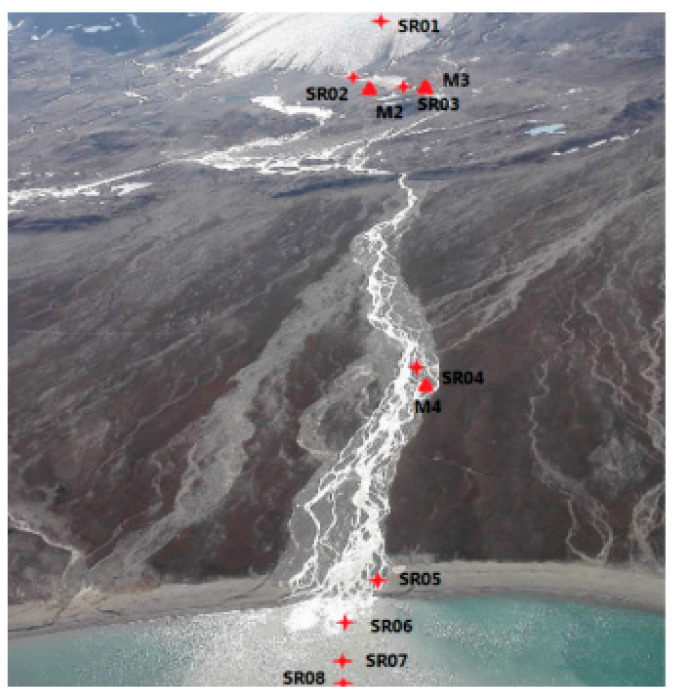
Sampling sites in Ny-Ålesund, Svalbard, Red symbols present sampling sites.

Ny-Ålesund, called a “glacier museum”, is located at the west of the Svalbard Archipelago (74~81° N, 10~35° E, Figure 2). China’s first Arctic station, i.e., the Yellow River Station, is located at Ny-Ålesund. Continued Arctic warming has led to rapid glacial melting and retreat, with an area reduction of about 512 km^2^ in Svalbard between 1961 and 1993. There was a total rainfall of higher than 65 mm in January 2018 in Ny-Ålesund, where the Kongsbreen Glacier has retreated by 5 km in the past 30 years [9]. The land glacier front has also retreated with the increasing surface runoff, which carries amounts of terrigenous materials into Kongsfjorden, a coastal fjord located in the northwest of Ny-Ålesund (Figure 2). Along with glacial melting and permafrost thawing, greenhouse gases accelerate emissions. This changes glaciers, their melting water and the permafrost and soil by which the melts flow. This certainly changes the microbial community structures and functions [3]. Such changes are extremely significant in summer, as shown by the increasing surface water in Kongsfjorden [5,9]. There are many studies on the prokaryotic [10,11,12,13,14,15] and eukaryotic communities [3,5,16,17,18,19,20,21,22] in Kongsfjorden. Proteobacteria and Bacteroidota are usually dominant in the prokaryotic communities [10,11,12,13,14,15]; Chlorophyta, Chrysophyta and dinoflagellates are usually predominant in the eukaryotic communities [3,5,14,15,16,17,18,19,20,21,22]. Comparatively, studies on microbial communities in both glacial water [23] and soil [24,25,26,27] were much fewer. Moreover, almost all of them were about prokaryotes, and the samplings were from different years. Consequently, these studies are scattered, and we cannot obtain a uniform pattern on how the microbes dynamically respond to the changing environments, which include pure glacial meltwater, glacial melting lakes, the soils by which glacial water flows, estuarine and seawater. This is a great gap in understanding the microbial response to Arctic warming. Our study exactly fills this gap by studying the microbial community’s biodiversity and structure, and their environmental correlations with the environmental changes in summer, which is the season of rapid glacier melting. This study is very distinctive and consolidated. It is a direct observation of microbial changes along with environmental changes. Prokaryote and eukaryote communities were both studied and compared in different types of soils and glacial melt-related waters. From this, we wondered whether there are distinct microbes in different environments, which microbes better adapt to the changing environments and to what degree environmental factors may explain the microbial community structures.

## 2. Material and Methods

Water samples from 12 sites (Table 1) from the Midre Lovénbreen glacier [10] to coastal water, i.e., Kongsfjorden, along the glacial melt were directly collected into TWIRL’EM sterile sampling bags (Labplas, Sainte-Julie, QC, Canada) on 5 and 7 July 2014. One liter of water was collected from each site and filtered through a 20 μm mesh and then a 0.2 μm pore-size membrane filter (Pall Corporation, New York, NY, USA). The microbial samples were then collected by filtering each water sample [28]. At the same time, three soil samples with their respective triplets were collected from different surface soils (0–5 cm) (Table 2); each sample was about 50 g [26]. All soil samples were also placed into TWIRL’EM sterile sampling bags. All filters and soil samples were stored in −80 °C in the Marine Laboratory of Kings Bay, frozen and sent back to China to analyze the biodiversity and structure of both the eukaryotic and prokaryotic communities at TinyGene Biotechnology Shanghai Co., Ltd. (Shanghai, China) [5,17].

### 2.1. Sampling and Environmental Factor Analyses

The temperature and salinity of the 12 water samples were measured by a WTW thermosalinometer (Table 1). The pH of 9 soil samples was measured by adding 10 mL of distilled water to 4 g of soil and recording the pH using a pH electrode (PHS-3C, Shanghai REX Instrument Factory, Shanghai, China) (Table 2). Ammonium nitrogen (NH_4_^+^), silicate silicon (SiO_4_^2−^), nitrite nitrogen (NO_2_^−^) and nitrate nitrogen (NO_3_^−^) were measured using a high-performance microflow analyzer (QuAAtro, SEAL Analytical GmbH, Norderstedt, Germany) [5].

### 2.2. Biodiversity and Taxonomy of Microbes (<20 μm) by Miseq Sequencing

DNA extraction and PCR amplification of rRNA genes is described in detail in Zhang et al. [5] and Cao et al. [10]. The Miseq platform with a 2 × 300 cycle V3 kit was used for sequencing after quantitation. The V3-V4 region of the 16S rRNA gene was amplified using the primers 926R (5′-CCGTCAATTCMTTTGAGTTT-3′) and 515F (5′-GTGCCAGCMGCCGCGGTAA-3′), and the V4 region of the 18S rRNA gene was amplified using the primer 3NDf (5′-GGCAAGTCTGGTGCCAG-3′) and its reverse primer. All singletons were removed from the prokaryotic and eukaryotic sequences. Moreover, sequences belonging to Metazoa were also removed from the eukaryotes (<20 μm). Finally, high-quality eukaryotic and prokaryotic sequences were processed as follows: using the furthest algorithm, eukaryotic and prokaryotic sequences were clustered, respectively, into OTUs (number of operational taxonomic unit) at 98% and 97% similarities [5,10]. R (Version 3.6.1) was used to construct the alpha-diversity indices (ACE and Shannon), Good’s coverage and so on. The data for the *α*-diversity analysis were normalized before use. Variations in the alpha-diversity of the groups of samples were evaluated using the Spearman rank analysis [5,10]. Taxonomy was assigned to the OTUs using the Silva database (Version 138, Bremen, Germany). Wang’s method with a confidence threshold of 80% was used according to Pruesse et al. [28]. The community diversities and similarities among all of the water samples were also analyzed with a 97% similarity in Perl and Mothur. All samples were normalized to analyze the Jaccard similarity (β-similarity). The sequence data were submitted to the NCBI Sequence Read Archive (SRA) database with the number SRP115724.

### 2.3. Statistical Analysis between Microbial Community and Environmental Factors

One-way ANOVA was used to determine whether there are any significant differences among the alpha-diversity of prokaryotes and eukaryotes in water and soil [5,10]. The relations between all prokaryotic OTUs and their corresponding environmental factors, including the pH, silicate and nitrogen (sum of nitrate, nitrite and ammonia in Table 2) in soil as well as the altitude, temperature and salinity in water, were analyzed using a canonical correspondence analysis (CCA, Canoco for Windows 4.5 software, Cambridge, UK). Comparatively, a redundant analysis (RDA, Canoco for Windows 4.5 software, Cambridge, UK) was used to analyze the environmental–eukaryotic relationships in both soil and water samples. A detrended correspondence analysis (DC) was used for the selection of a CCA or RDA [29]. The largest DC axial length, 3, is the selection boundary for the CCA and RDA. A CCA is selected if the length is >4, and an RDA is selected if the length is <3. Both a CCA and RDA can be used if the length is between 3 and 4. Based on this criterion, relationships between prokaryotic and eukaryotic OTUs and their corresponding environmental factors were analyzed by a CCA and RDA, respectively. A Pearson analysis of the different environmental factors and microbial OTUs was also performed by a CCA or RDA at the same time [5,17].

## 3. Results and Discussion

### 3.1. Environmental Description of Sampling Sites

The water sampling sites were along a glacial melt from the Midre Lovénbreen glacier, a typical land glacier at an elevation of about 800 m. A large amount of meltwater always forms during the melting period [10]. The runoff from the glacier flows out of a sub-glacial environment and crosses the permafrost and thawed soil until it reaches coastal water. There is an estuary where glacial meltwater meets seawater. The temperature gradually increased from −1 to 7 °C from the glacial to the coastal seawater with salinity changes from 0 to 38 (Table 1). This indicates increases in both temperature and salinity with increasing runoff. There were obvious gradients of temperature, salinity and latitude in our sampling sites. The soils were rich in nutrients and alkaline (Table 2) [23,30,31]. The nutrients in glacial melts were constantly changing as meltwater flowed through different permafrost and soil sites [23,32].

### 3.2. Microbial Community Diversity and Composition by High-Throughput Sequencing

**Figure 3:**
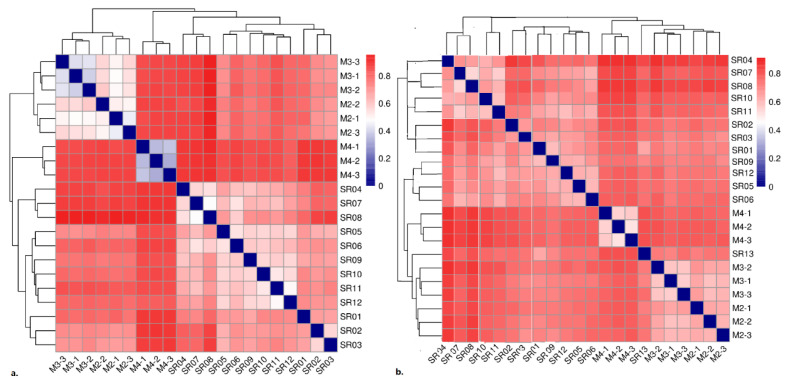
Jaccard similarity analysis based on OTUs at different sampling sites: (**a**) prokaryotic community; (**b**) eukaryotic community. Color bar and clustering tree indicates similarity among ß-diversity at different sampling sites; the similarity was calculated by Euclidean distance and furthest distance between groups.

Along with the environmental changes, the microbial community changes significantly, whether eukaryotes or prokaryotes (Figure 3). The closer the sampling sites were, or the closer the physical and chemical properties of sampling sites were, the more similar the prokaryotic and eukaryotic microbial communities were. The microbes in the soil were much more similar than those in the water. Both prokaryotes and eukaryotes have their most distinctive community diversities at the highest latitude.

#### 3.2.1. Community Diversity and Composition of Prokaryotes

Good’s coverage estimator of the prokaryotic OTUs in the samples ranged from 98.60% to 99.56% (Table 3). This indicates that the sequencing is deep enough. A total of 972,163 sequences (reads) and 10,037 OTUs (at 97% similarity) were identified for the prokaryotic community. The sequence number of each sample ranged from 23,095 (SR04) to 78,053 (M4-3) from which between 238 (SR08) and 649 (M4-3 and SR05) OTUs were recognized. There were significant differences between the Shannon index (*p* = 0.007), which is an indicator of biodiversity, and OTUs (*p* = 0.0082). There were relatively few types of species, as shown by the number of OTUs and the minimum biodiversity at the pure glacial water site SR01. Comparatively, the maximum OTUs and biodiversity were lowest at the soil site (M4-3). There were distinct differences among the microbial communities in soil and water, as well as among different soil sampling sites at different altitudes, obtained by a Jaccard similarity analysis (Figure 3). Generally, species from different soils were much more similar than those from water. Species from pure glacier-related water samples, including glacial water (SR01), subglacial soil water (SR02) and subglacial lake water (SR03), were distinctly different from those from estuary water (SR04–SR12). SR04 was especially distinct as it was taken from glacier water that runs directly into the estuary without contact with soils. However, in the estuary water, the microbial communities from closer latitudes were much more similar than those from glacier water or seawater.

**Figure 4:**
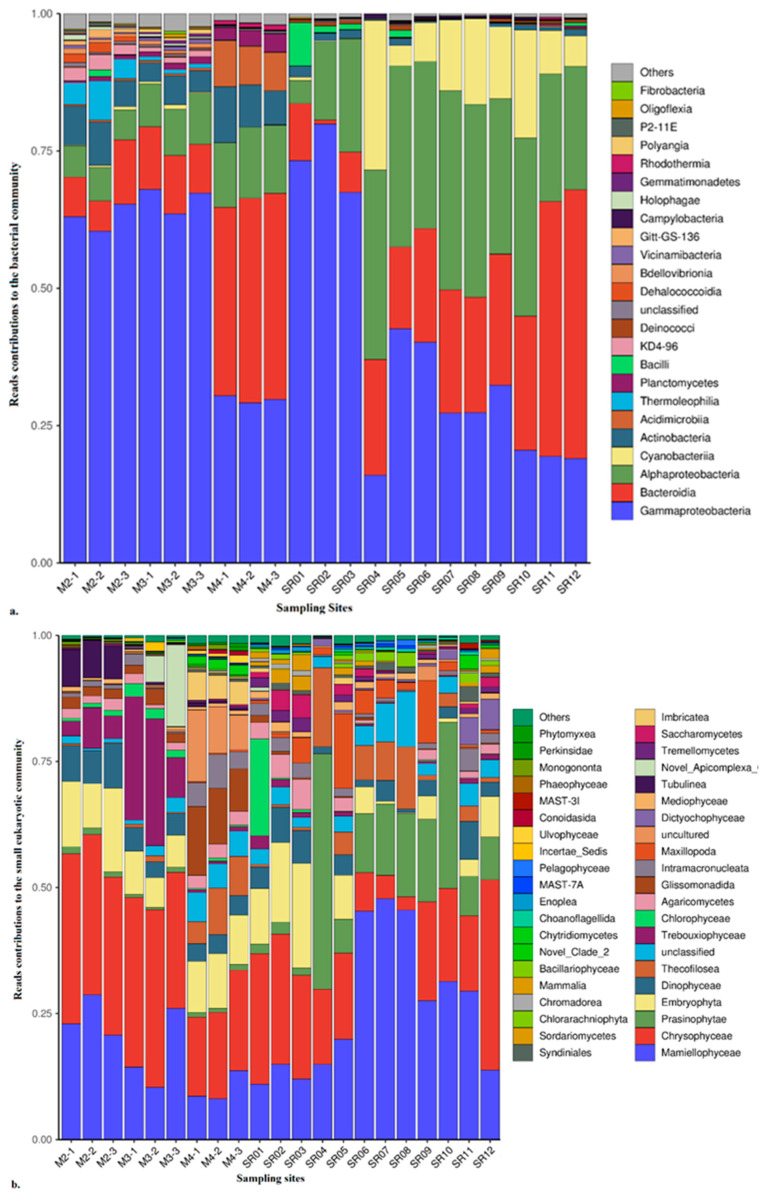
Reads of contributions to the bacterial library of different classes at different sites in Ny-Ålesund. M2–M4 indicate soil sites with their respective triplicates; SR01–SR12 indicate different sites of glacial melts: (**a**) for bacterial community, (**b**) for eukaryotes < 20 μm.

Proteobacteria, Bacteroidota, Cyanobacteria, Actinobacteriota, Chloroflexi, Planctomycetota, Firmicutes and Acidobacteriota were the main bacterial phyla; their respective contributions to the whole bacterial DNA library were 41.5~94.2%, 0.8~49.0%, 0~27.3%, 0.1~18.7%, 0~6.8%, 0~3.7%, 0~8.4% and 0~2.0%. Their respective averages were 63.7%, 20.5%, 5.6%, 5.3%, 1.1%, 0.8%, 0.7% and 0.5%. The community compositions at the phylum level were very similar to those from similar sampling sites [30,31,32,33,34], ancient Siberian permafrost [32] and the northeast Eurasian Austrian Alps (10.162–13.278° E, 46.778–47.135° N and 2100~2880 m above sea level) [34]. The community biodiversities in this study were much more than those in the Antarctic soils [26,32] and Yunnan–Kweichow Plateau [24], where Proteobacteria, Actinobacteria, Acidobacteria, Bacteroidota and Verrucomicrobia can account for about 90% of all bacterial phyla. Gammaproteobacteria, Bacteroidia, Alphaproteobacteria, Cyanobacteria, Actinobacteria, Acidimicrobiia, Thermoleophilia, Planctomycetes, Bacilli and KD4-96 (Chloroflexi) were the main bacterial classes, and their respective contributions to the whole bacterial DNA library were 16~79.9%, 0.7~48.9%, 4.2~36.2%, 0~27.3%, 0.1~8.4%, 0~7.1%, 0~3.3%, 0.7~7.9% and 0~2.8%. Their respective averages were 44.9%, 20.3%, 5.5%, 3.2%, 1.2%, 0.8%, 0.7%, 0.7% and 0.5% (Figure 4a). Comparatively, there are 44 and 22 genera that have average contributions of ≥0.5% and >1% to the DNA library, respectively. Significant differences (<0.5) were found among all the major classes. The genera with contributions of >1% were *Escherichia-Shigella*, *Rhodoferax*, Sulfitobacter, *Clade_Ia*, *Thiobacillus*, *Polaromonas*, *Flavobacterium*, *Polaribacter*, *Stenotrophomonas*, *Phyllobacterium*, *Sediminicola*, *Ulvibacter*, *Acinetobacter*, *NS5_marine_group*, *Granulosicoccus*, *Pseudoalteromonas*, *SAR92_clade*, *Paraperlucidibaca*, *Methyloversatilis*, *Empedobacter* and *Massilia*.

#### 3.2.2. Community Diversity and Composition of Eukaryotes

Good’s coverage estimator of the eukaryotic OTUs in the samples ranged from 99.07% to 99.74% (Table 4). This indicates that the sequencing is deep enough. A total of 1,085,257 sequences (reads) and 9188 OTUs (at 97% similarity) were identified for the eukaryotic community. The sequence number of each sample ranged from 35,130 (SR08) to 114,701 (M3-2) from which between 223 (SR04) and 695 (M4-3) OTUs were recognized with a 98% similarity. There were very significant differences among the biodiversity as indicated by the Shannon index (*p* = 0.0065) and OTU numbers (OTUs, *p* = 0.0074). The minimum types of species (OTUs) and biodiversity were at the glacial runoff into the estuary water (SR04). Comparatively, the maximum types of species and the relatively large biodiversity were lowest at the soil site (M3-2). The eukaryotes were more confused than the prokaryotes in this estuarine mixture. This may indicate that eukaryotes are better adapted to changing environments than prokaryotes. Therefore, the prokaryotes had a higher number of species (Chao1) than the eukaryotes (<20 μm) at the same sampling site (Table 3 and Table 4). Comparatively, the α-diversities (Shannon index) were not so distinctly different.

Chlorophyta, Chrysophyta, Cercozoa, Dinoflagellata, Basidiomycota, Ciliophora, Ascomycota, Diatomea, Apicomplexa and Protalveolata were the main eukaryotic phyla, and their respective contributions to the whole small eukaryotic DNA library were 10.1~64.6%, 2.6~37.7%, 1.5~41%, 0.7~8.9%, 0~9%, 0.2~6.7%, 0~7.6%, 0~2.9%, 0~16.2% and 0~3%. Their respective averages were 37.4%, 21.7%, 10.2%, 4.3%, 2.8%, 1.9%, 1.5%, 1.2%, 1.2% and 0.7%. There were five classes with respective contributions to the whole eukaryotic DNA library of ≥0.5% and also of ≥1%, i.e., Mamiellophyceae, Chrysophyceae, Prasinophytae, Trebouxiophyceae, Chlorophyceae and Tubulinea (Figure 4b). Their respective contributions were 8.1~47.8%, 2.6~37.7%, 0~25.2%, 0~19.2% and 0~7.3%. Their respective averages were 37.4%, 22.2%, 8.4%, 4.0% and 1.5%. Very significant differences (<0.01) were found among all of the major classes. Comparatively, there are 12 genera that have average contributions of ≥0.5% to the DNA library. They are *Micromonas* (Mamiellophyceae), *Poterioochromonas* (Chrysophyceae), *Karlodinium* (Dinophyceae), *Paraphysomonas* (Chrysophyceae), *Heteromita* (Glissomonadida), *Cryothecomonas* (Thecofilosea), *BOLA868* (Tubulinea), *Prasinoderma* (Prasinophytae), *Bathycoccus* (Mamiellophyceae), *Protaspis* (Thecofilosea), *Saitozyma* (Tremellomycetes) and *NOR26* (Chlorarachniophyta). Their respective contributions to the whole DNA library were 7.7~40.8%, 0.1~31.8%, 0.4~7.1%, 0.1~23.7%, 0~13%, 0~8%, 0~7.3%, 0~1.9%, 0.1~4.4%, 0~3.2%, 0~2.7% and 0~2.8%, and their respective averages were 20.3%, 14.7%, 2.9%, 2.8%, 2.2%, 1.2%, 1.1%, 0.9%, 0.9%, 0.8%, 0.7% and 0.5%. Consequently, there were seven genera with contributions of > 1%. This is in accordance with the study in [35], i.e., the nanoplankton and picoplankton are predominant in Arctic waters. Chlorophyta, Chrysophyta and dinoflagellata are mixotrophic, which enhance the transfer of biomass to large-sized classes further up the food web. This results in an approximately three-fold increase in organism size and an approximately 35% increase in sinking carbon flux [35]. Phagotrophy is effective for these flagellates in surviving polar nights. *Micromonas* and *Poterioochromonas* were dominant, respectively, in the Kongsfjorden in the years 2012 and 2013 [5,17]. Comparatively, Dinophyceae were predominant in 2015 [3].

#### 3.2.3. General Discussion for Prokaryotes and Eukaryotes in Ny-Ålesund, Svalbard

Although our sampling sites were distinct, we found few changes in the dominant groups, which were basically globally dominant groups, such as Proteobacteria and Bacteroidota in the prokaryotes and Chlorophyta and Chrysophyta in the eukaryotes. Proteobacteria and Bacteroidia were the most abundant prokaryotic phyla, and Chlorophyta and Chrysophyta were the most abundant eukaryotic phyla in both soil and water. They were all documented as dominant in Kongsfjorden, the coastal water in Ny-Ålesund [3,5,10,17,18,19,20,21,22,27]. Comparatively, the microbial population changes greatly at class and lower levels with changes in their growth media (Figure 1). Gammaproteobacteria and Chrysophysceae were the most abundant in the soil stations, whereas Alphaproteobacteria and Bacteroidia were the most abundant prokaryotes and Mamiellophyceae and Prasinophytae were the most abundant eukaryotes in the water stations. Bacilli and Chlorophyceae were only found in glaciers. Actinobacteria, KD94-96, Thermleophilia, Embryophyta, Trebouxiophyceae and Sordariomycetes were special microbes in soil. Dinoflagellates are highly adaptable to environments and can be found to contribute to the eukaryotic DNA libraries in all our studied media. Cyanobacteria were terrestrially originating as shown by the maximum contributions at the station SR04, a site on glacial runoff, and can adapt to both glacial and sea waters as they had relatively large contributions in the estuary (SR05–SR08 and SR09–SR12). This is different from the study [22], where the Cyanobacteria were from soil. However, there is a 5-year time lag between our study and theirs. This was an accelerated Arctic warming period, so the microbial community’s structure has already changed with climate change according to their environmental sensitivity [3,5,10,17,18,19,20,21,22,27]. The prokaryote and eukaryote communities were distinct between soil and water (Table 3 and Table 4). Biodiversity (Shannon index) and species (OTUs) in the soil increased along with a decrease in altitude. Pure glacial water-related stations (SR01–SR04) have the most special microbial community [36]. Comparatively, there were no distinct differences in the biodiversity and species of the microbial community in mixed water, whether seawater in the estuary (SR05–SR07) or glacial melt in the estuary (SR08–SR10). Gammaproteobacteria were gradually replaced by Alphaproteobacteria and Bacteroidia along with glacial water that runs into the coastal water [10,33]. Chrysophyceae and Embryophyta were replaced by Mamiellophyceae and Prasinophytae [5]. That is, Chrysophyta is better adapted to fresh water, while Chlorophyta is better adapted to seawater. Moreover, flagellates, rather than diatoms, contributed more to the carbon in the Arctic [12,14,15,19,20,21,22]. The prokaryotic biodiversity was much greater than the eukaryotic biodiversity at all of the sampling sites. However, only the numbers of prokaryotic species (OTUs) were more than those of the eukaryotes at a high altitude (*p* < 0.01). There were no significant differences between the OTUs at a lower latitude (*p* > 0.5), regardless of the medium.

Among the abundant genera with contributions of > 1% to the prokaryotic DNA library, Sulfitobacter (Alphaproteobacteria), *Polaromonas*, *Rhodoferax*, *Thiobacillus*, *Pseudoalteromonas* and *Massilia* (Gammaproteobacteria), *Flavobacterium*, *Polaribacter*, *Ulvibacter* and *Empedobacter* (Bacteroidia) were the dominants in the Kongsfjorden [10,11,12,14,15]. *Clade_Ia* and *Phyllobacterium* (Alphaproteobacteria) were from a glacier as they only had contributions in water. They lived well in the mixed water of glacier runoff and sea, as shown by the relatively large contributions in the estuary. *Stenotrophomonas*, *Granulosicoccus* and *Paraperlucidibaca* (Gammaproteobacteria) were, respectively, from soil at high and low altitudes, as they had distinct, large contributions at their corresponding sampling sites. *Stenotrophomonas* can live in glacier water, but *Granulosicoccus and Paraperlucidibaca* cannot live in water, as shown by their respective contributions in different types of water. *SAR92_clade* (Gammaproteobacteria) were certainly from a glacier and can live in mixed glacier and sea water, as shown first at SR01, the pure glacier runoff sampling site; it had the largest contribution at SR04, the estuary water with the largest proportion of glacier runoff. *SAR92_clade* gradually decreased from the glacier to coastal water (Figure 1). Comparatively, *Methyloversatilis* can only live in pure glacier water. *NS5_marine_group* (Flavobacteriaceae) were from seawater. Their contribution to the prokaryotic library was larger when the proportion of seawater to estuary water was larger [3,10,23].

As for the abundant genera with contributions of ≥0.5% to the eukaryotic DNA library, *Micromonas and Bathycoccus* (Mamiellophyceae), *Poterioochromonas* and *Paraphysomonas* (Chrysophyceae), *Karlodinium* (Dinophyceae), *Heteromita* (Glissomonadida), *Cryothecomonas* and Protaspis (Thecofilosea), *Prasinoderma* (Prasinophytae) and *NOR26* (Chlorarachniophyta) were common in Kongsfjorden [3,17,18,19,20,21,22,26,37,38]. *BOLA868* (Tubulinea) was special at the high-latitude soil sampling site (M2) (Figure 4). *BOLA868* sharply decreased at a lower altitude and cannot survive in water. Undoubtedly, *Saitozyma* (Tremellomycetes) was from a glacier, as shown by its high contributions in glacier water-related sampling sites, i.e., SR01–SR03. It also slightly contributed to the eukaryotic DNA libraries in both soils and estuaries, by which glacial meltwater has flowed or into which it has run. However, it cannot survive in pure seawater. As analyzed above, cyanobacteria and dinoflagellates are more abundant in coastal waters, where the glacial water increases with the temperature.

### 3.3. Environmental Correlations of Microbes in Ny-Ålesund

#### 3.3.1. CCA for Prokaryotic Communities

**Figure 5:**
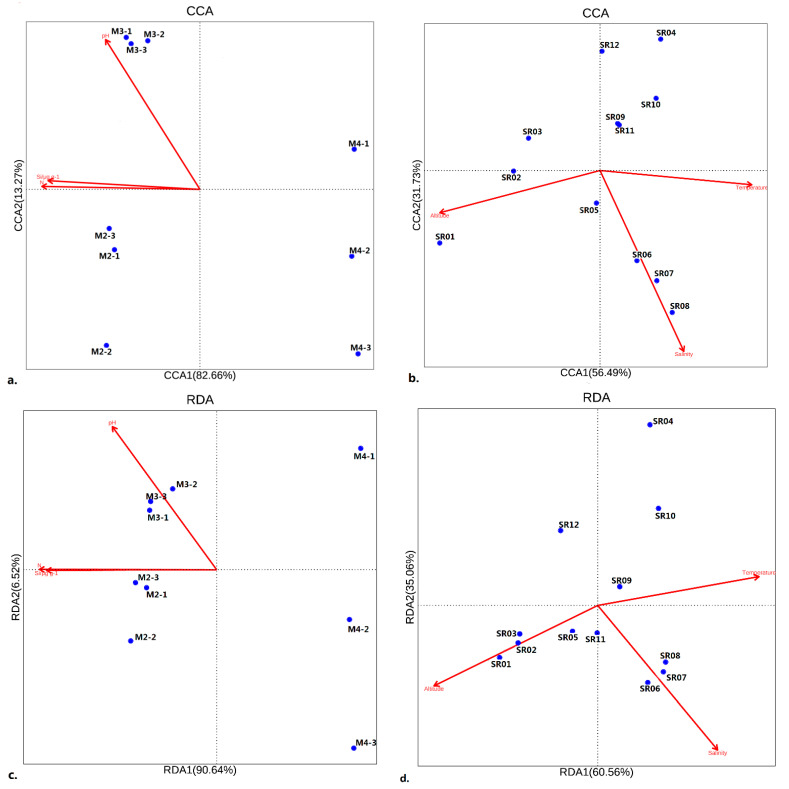
Relationships of microbial community structure at different sampling sites with physiochemical factors in an ordination diagram with the first two axes of the canonical correspondence analysis (CCA) and redundant analysis (RDA). Red arrows with different lengths denote relative correlations of different independent variables with the community structure factors: (**a**,**b**) for prokaryote communities in soil and water samples, respectively; (**c**,**d**) for eukaryote communities in soil and water samples, respectively.

Temperature, macro-nutrients and other abiotic factors all can affect a microbial community’s structure [5,10,17,18,19,20,21,22,27,38,39]. Nitrogen, pH and silicate (Table 2) were all important to the soil prokaryotic community [12,16,26,27], as the environmental–biological relationship can be as high as 97.87%. The first two axes accounted for 95.93% of the whole relationship (Figure 5a). Among this, nitrogen, silicate and pH accounted for 38.15%, 6.15% and 2.48%, respectively. However, only the prokaryotic community at the highest altitude (M2) was significantly affected by the three abiotic factors, which have almost no effect on the microbial communities at the two other lower altitudes. Comparatively, salinity, altitude and temperature also accounted for a large proportion of the prokaryotic community in water. The first two axes accounted for 88.22% of the whole relationship. Among this, altitude, temperature and salinity accounted for 28.27%, 10.86% and 8.24%, respectively. Similarly, the prokaryotes at different water sampling sites can be classified into distinct groups according to the water’s characteristics (Figure 5b). Pure glacier water-related samples (SR01) were mainly affected by altitude. Samples from the seawater in the estuary (SR06–SR08) were mainly affected by salinity and temperature, whereas mixed water samples with a high proportion of glacier water (SR02, SR03, SR04 and SR09–SR12) were almost unaffected by the three environmental factors.

#### 3.3.2. RDA for Eukaryotic Communities

RDA was more appropriate for analyzing the environmental correlations of the eukaryotic communities in soil and water (Figure 5c,d). Comparatively, eukaryotes were less affected than prokaryotes by environmental factors. Nitrogen, silicate and pH were also important to the soil eukaryotic community, as they accounted for 90.83% of the community structure. The first two axes accounted for 97.16% of the whole relationship (Figure 5c). Among this, nitrogen, silicate and pH accounted for 26.65%, 12.78% and 8.66%, respectively. Similar to the prokaryotes, the eukaryotic community at the highest altitude was still the most distinct compared with those at lower latitudes. However, no distinct environmental correlations were found in the entire soil community. Comparatively, altitude, salinity and temperature also accounted for a large proportion of the eukaryotic community in water (Figure 5d). The first two axes accounted for 95.62% of the whole relationship. Among this, altitude, salinity and temperature accounted for 25.77%, 17.72% and 3.46%, respectively. Similarly, eukaryotes at different water sampling sites can be classified into distinct groups according to the water’s characteristics. Pure glacier water-related samples (SR01–SR03) were mainly affected by altitude. Samples from the seawater in the estuary (SR05–SR08) were mainly affected by salinity and temperature, whereas mixed water samples with a high proportion of glacier water (SR04, SR09–SR12) were very weakly affected by temperature.

## 4. Conclusions

The microbial community in pure glacier melts was the most distinct. The species from glacier-related water were distinctly different from those in glacial melt and coastal water mixtures. Eukaryotes were more stable than prokaryotes in changing environments. Proteobacteria, Bacteroidota, Chlorophyta and Chrysophyta were the dominants in both soil and water. Dinoflagellates were highly adaptable to all environmental types. Cyanobacteria were terrestrial and can adapt to both glacial and sea waters. Altitude had significant correlations with prokaryotic and eukaryotic species. Cyanobacteria and dinoflagellates will be more abundant in coastal water in the future.

## Figures and Tables

**Table 1:** Water sampling information at different sites.

Sampling Sites	Latitude and Longitude	Altitude/m	Temperature/°C	Salinity
SR01 (Glacial water)	78°53′40.88″ N 12°02′38.67″ E	78	−1.0	0
SR02 (Subglacial soil water)	78°53′40.85″ N 12°02′33.15″ E	58	0.0	0
SR03 (Subglacial lake water)	78°53′51.36″ N 12°02′50.25″ E	21	5.0	0
SR04 (Glacial runoff into estuary water)	78°54′51.18″ N 12°02′29.93″ E	0	6.0	0
SR05 (Sea water in estuary #1)	78°54′52.50″ N 12°02′29.70″ E	0	6.0	17
SR06 (Sea water in estuary #2)	78°54′53.42″ N 12°02′29.34″	0	6.5	27
SR07 (Sea water in estuary #3)	78°54′55.07″ N 12°02′29.01″ E	0	7.0	36
SR08 (Sea water in estuary #4)	78°54′56.43″ N 12°02′28.64″ E	0	7.0	38
SR09 (glacial melt in estuary #1)	78°54′53.10″ N 12°02′35.70″ E	0	4.5	0
SR10 (glacial melt in estuary #2)	78°54′53.42″ N 12°02′38.34″ E	0	4.5	0
SR11 (glacial melt in estuary #3)	78°55′55.07″ N 12°02′37.51″ E	0	5.0	0
SR12 (glacial melt in estuary #4)	78°54′58.47″ N 12°02′35.15″ E	0	5.0	0

**Table 2:** Soil sampling information at different sites.

Sample		Latitude and Longitude	pH ± S.D.	NH_4_^+^ ± S.D. (μg/g)	SiO_4_^2−^ ± S.D. (μg/g)	NO_2_^−^ ± S.D. (μg/g)	NO_3_^−^ ± S.D. (μg/g)
M2	M2-1	78°53′40.85″ N 12°02′33.15″ E	8.55 ± 0.03	1.56 ± 0.06	10.36 ± 0.01	0.33 ± 0.02	0.30 ± 0.02
	M2-2		8.55 ± 0.03	1.96 ± 0.05	10.62 ± 0.01	0.32 ± 0.02	0.31 ± 0.02
	M2-3		8.55 ± 0.03	1.85 ± 0.04	10.56 ± 0.01	0.31 ± 0.02	0.30 ± 0.02
M3	M3-1	78°53′51.36″ N 12°02′50.25″ E	8.69 ± 0.04	1.31 ± 0.06	10.67 ± 0.01	0.20 ± 0.03	0.23 ± 0.03
	M3-2		8.50 ± 0.02	1.37 ± 0.04	7.63 ± 0.03	0.18 ± 0.03	0.21 ± 0.03
	M3-3		8.70 ± 0.02	2.28 ± 0.02	10.19 ± 0.01	0.17 ± 0.02	0.25 ± 0.03
M4	M4-1	78°54′51.18″ N 12°02′29.93″ E	8.58 ± 0.01	1.74 ± 0.05	6.56 ± 0.03	0.16 ± 0.02	0.64 ± 0.01
	M4-2		8.56 ± 0.04	1.26 ± 0.06	9.66 ± 0.02	0.13 ± 0.03	0.73 ± 0.01
	M4-3		8.35 ± 0.05	1.58 ± 0.04	6.67 ± 0.03	0.18 ± 0.02	0.79 ± 0.01

**Table 3:** Summary information from high-throughput sequencing data for the 21 bacterial samples.

	Coverage (%)	Reads	OTUs	ACE	Shannon
M2-1	99.16	44,063	508	660.47	3.97
M2-2	99.21	46,998	428	589.03	3.70
M2-3	99.03	40,992	482	686.26	3.67
M3-1	98.95	49,590	603	794.23	4.01
M3-2	98.91	48,208	627	821.67	4.26
M3-3	98.91	49,815	588	796.23	4.04
M4-1	98.98	55,531	560	771.87	4.44
M4-2	99.01	53,353	609	783.71	4.68
M4-3	98.91	78,053	649	842.38	4.71
SR01	99.33	25,870	313	470.04	3.04
SR02	99.03	73,053	402	643.31	3.12
SR03	99.25	71,130	347	515.29	3.09
SR04	99.33	23,095	274	500.28	3.29
SR05	98.60	77,477	649	977.53	4.33
SR06	99.13	27,951	424	610.93	3.89
SR07	99.29	27,049	329	526.63	3.50
SR08	99.56	23,616	238	339.76	3.36
SR09	98.98	25,251	465	711.80	3.82
SR10	98.79	29,946	499	832.23	3.83
SR11	98.93	29,691	489	718.61	3.59
SR12	98.80	71,431	554	834.28	3.71
*F*-test	0.06	0.013	0.0082	0.024	0.007

**Table 4:** Summary information from high-throughput sequencing data for the 21 eukaryotic samples.

	Coverage (%)	Reads	OTUs	Chao1	Shannon
M2-1	99.64	56,520	330	378.88	2.74
M2-2	99.47	63,412	295	400.32	2.64
M2-3	99.54	78,284	342	410.36	2.87
M3-1	99.44	98,071	357	449.97	2.75
M3-2	99.39	114,701	328	449.39	2.93
M3-3	99.47	59,915	326	417.67	2.61
M4-1	99.38	48,574	611	689.91	4. 10
M4-2	99.42	51,680	641	703.65	4.23
M4-3	99.30	71,068	695	774.08	4.18
SR01	99.28	38,693	644	730.26	3.71
SR02	99.74	39,070	419	453.03	3.64
SR03	99.54	37,452	428	499.94	3.52
SR04	99.56	38,194	223	314.74	2.43
SR05	99.45	36,135	501	571.00	3.83
SR06	99.27	35,166	442	604.56	3.36
SR07	99.21	35,580	444	605.79	3.22
SR08	99.31	35,130	342	502.67	3.09
SR09	99.07	37,275	515	677.11	3.14
SR10	99.45	36,202	317	426.31	2.79
SR11	99.44	36,203	501	608.00	4.06
SR12	99.39	37,932	487	580.94	3.56
*F*-test	0.10	0.025	0.0074	0.034	0.0065

## Data Availability

The sequence data are deposited in the National Center for Biotechnology Information Sequence Read Archives (SRA) under the number SRP115724.
